# Commentary on ‘Exclusion rates in randomized trials of treatments for physical conditions: a systematic review’

**DOI:** 10.1186/s13063-021-05019-9

**Published:** 2021-01-21

**Authors:** Anna Ugalde, Nicole Kiss, Patricia M. Livingston, Nicole Rankin

**Affiliations:** 1 School of Nursing and Midwifery and Institute for Health Transformation, Deakin University, 1 Gheringhap Street, Geelong, VIC Australia; 2 Deakin University, Institute for Physical Activity and Nutrition (IPAN), Geelong, Australia; 3 Allied Health Research, Peter MacCallum Cancer Centre, Melbourne, Australia; 4 Research in Implementation Science and e-Health (RISe) Faculty of Medicine and Health, The University of Sydney, Camperdown, NSW Australia

We read with interest the paper by He, Morales and Guthrie [1] recently published in *Trials*. This systematic review reports that 50 randomised controlled trials for people with physical conditions excluded the majority of potential patients, with a median exclusion rate of 77.1% of potential participants, with only 5.2% of trails excluding less than 25%. This review adopted high-quality systematic review procedures, including two reviewers performing data screening and extraction. As the authors reflect, the findings present significant issues for the generalisability of the studies’ results.

Systematic reviews aim to synthesise an existing body of literature with a predetermined and structured method, often focusing on identifying the efficacy of similar interventions and comparing outcomes [2]. Systematic reviews are typically relied upon as a source to identify those interventions that may be most effective in clinical practice. He et al. focused on the exclusion rates of physical condition randomised controlled trials, rather than trial efficacy, which enables important reflections to be made upon the design and conduct of these studies. The samples that participate in randomised controlled trials are important to consider, as trials can lack external validity, with a small, select group of participants participating in a trial that may lack relevance to broader patient populations [3]. He, Morales and Guthrie reflect that their review demonstrated the narrow populations selected to participate in trial studies, with samples that have a higher chance of improving with treatment, and a lower chance of adverse events.

In considering real-world effectiveness of interventions, we reflect on the implementation science literature, in particular the work of Proctor and colleagues, who propose a set of outcomes that can be used to evaluate successful implementation of interventions [4]. Proctor et al. present eight implementation outcomes: acceptability, adoption, appropriateness, feasibility, fidelity, implementation cost, penetration and sustainability. These outcomes are distinguished from service outcomes (as defined by the Institute of Medicine [5] such as timeliness, efficiency and equity) or client outcomes (such as satisfaction or function). The feasibility implementation outcome is a reflection of whether new program can be successfully used within a setting, noting that it is ‘reflected in poor recruitment, retention or participation rates’ [4]. The findings presented by He, Morales and Guthrie are also a reflection of the limited feasibility of trials for treatments for physical conditions given the significant exclusion of participants.

We recently operationalised Proctor et al.’s implementation outcomes to report on the potential of implementing cancer caregiving interventions in practice, concluding that such studies lack sufficient detail in both design and reporting to sufficiently bridge the evidence to practice gap [6]. For the feasibility outcome, we applied an operational definition as ‘participation of caregivers, and time commitment to intervention delivery’. We found that less than one-third of eligible caregivers participated in those studies that met inclusion criteria.

There is significant opportunity to improve the reporting of efficacy trials to consider future interventions and outcomes about effectiveness and potential for implementation in real-world settings. Our example of operationalising implementation outcomes used clearly defined criteria to enable measurement and systematic application across one aspect of cancer care [7]. The findings of He et al.’s study supports calls for greater use of pragmatic trial designs [8, 9]. There is a wealth of evidence about the significant challenges in improving healthcare outcomes and implementation science has a central role in addressing such challenges [10]. Indeed, there is a need to consider implementation early to conduct studies that have the most potential to be implemented [6].

While these authors highlight the limited generalisability of trials for physical conditions, we also consider that the results also indicate lack of feasibility for real-world impact. We note the many opportunities to document strategies to improve feasibility, including codesign of interventions [11] and conducting quality pilot studies [12]. We also suggest the need to broaden inclusion criteria of studies to enable greater participation from the population.

## Data Availability

Not applicable
